# A novel HIV model through fractional enlarged integral and differential operators

**DOI:** 10.1038/s41598-023-34280-y

**Published:** 2023-05-12

**Authors:** M. A. Barakat, Abd-Allah Hyder, Areej A. Almoneef

**Affiliations:** 1 Department of Computer Science, University College of Al Wajh, University of Tabuk, Tabuk, Saudi Arabia; 2 Department of Mathematics, Faculty of Sciences, Al-Azhar University, Assiut, Egypt; 3 Department of Mathematics, College of Science, King Khalid University, P.O. Box 9004, 61413 Abha, Saudi Arabia; 4 Department of Engineering Mathematics and Physics, Faculty of Engineering, Al-Azhar University, Cairo, Egypt; 5 Department of Mathematical Sciences, College of Science, Princess Nourah Bint Abdulrahman University, P.O. Box 84428, Riyadh, 11671 Saudi Arabia

**Keywords:** Computational biology and bioinformatics, Mathematics and computing

## Abstract

This article presents a novel mathematical fractional model to examine the transmission of HIV. The new HIV model is built using recently fractional enlarged differential and integral operators. The existence and uniqueness findings for the suggested fractional HIV model are investigated using Leray–Schauder nonlinear alternative (LSNA) and Banach’s fixed point (BFP) theorems. Furthermore, multiple types of Ulam stability (U-S) are created for the fractional model of HIV. It is straightforward to identify that the gained findings may be decreased to many results obtained in former works of literature.

## Introduction

Numerous dangerous infectious diseases are mostly brought on by bacteria and viruses. The impact of infectious illnesses on society is enormous. Approximately one-fourth of all mortalities globally are caused by these infections. HIV which induces immunodeficiency syndrome (AIDS) killed the lives of 36.3 million people, is still a significant global health issue. HIV specifically harms $\text {CD}4^+$ T-cells (CDFP-TC), which are the invulnerable system’s essential component and targets the human body’s immune system. The virus reduces the body’s defenses, making the affected person more vulnerable to other illnesses. HIV replicates and targets CDFP-TC when it enters the human body.

The HIV life cycle involves a number of stages^1^. First, once HIV connects to CDFP-TC receptors, the virus’ envelope starts to meld with the cell’s membrane. The virus may get into the cell at this stage, which is referred to as binding and fusion. Second, HIV uses the reverse-transcriptase enzyme to mutate its genetic code from RNA into DNA by releasing it from the CDFP-TC. Reverse transcription is the process that enables HIV to enter the CDFP-TC nucleus. Third, when HIV enters the nucleus of the CDFP-TC, it releases an extra enzyme known as integrase. To join the DNA of the CDFP-TC and the virus, the latter utilizes this enzyme. Even cutting-edge lab testing can’t detect the virus at this point since it is considered latent. This phase is known as integrating and transcription. Fourth, HIV may now be capable to employ the CDFP-TC mechanism to produce viral proteins since it has been incorporated into the latter’s DNA. At this time, HIV may also create more of its genetic material (RNA). It is possible that these two conditions will drive it to manufacture more virus particles. This phase is known as replication. Fifth, the newly created HIV proteins and RNA are transported to the CDFP-TC edge where they grow to unripe HIV. As those viruses are not yet contagious, this phase can be referred to as the assembly. Lastly, the unripe virus exits the CDFP-TC. The viral proteins are then modified by the release of the proteolytic enzyme, which makes the virus infectious. This phase is called upgrowth. Antiretroviral therapy (ART) is the use of HIV medications for treating HIV infection and protecting the impregnable system by preventing the virus shape from replicating at several phases of its cycle. For more details see^2–5^. A crucial technique for predicting the likelihood and severity of infections as well as understanding their dynamic behavior was the use of mathematical models and simulations. These models are useful instruments and essential for understanding the mechanics of the immunological respond to HIV’s infection. Readers are advised to read some literature as^6–9^.

Based on several earlier publications about the model of HIV, researchers frequently used differential equation systems to show how HIV and uninfected CDFP-TC are related, as well as how medication therapy affects infected cells. A straightforward model for HIV infection was put up in^10^ to study various dynamic characteristics of HIV infection of CDFP-TC. A mathematical coupled model for the initial progression of HIV of the first sort was put out by Tuckwell et al.^11^. Regardless of the numerical solvability of their model, nonlinear effects allow for general theoretical generalizations. Rong et al. used a model in^12^ to examine the initial limitations that might lead to viral resistance to antiretroviral medications. An initial HIV infection model has been proposed and looked at by Srivastava et al.^13^ during therapy. In their model, only reverse transcription inhibitors were included, and the pharmaceutical therapy model was built correctly. Moreover, the well-posedness of the HIV fractional model was proved by Jleli and Samet in^14^. They employed Grönwall’s formula and Perov’s theorem to acquire their results.

Numerous scholars have recently been studying the subject of fractional calculus, which has proved crucial to science and engineering. Recently, There have been applications of fractional calculus in many disciplines. Additional details on fractional calculus and its uses may be found in the sources^15–21^. Several academics looked into the numerical fractional estimates, equilibrium conditions, and disease-free stability for certain epidemic fractional models^22,26^. Using fractional calculus for examining HIV models has produced a number of different study conclusions. By employing fractional Caputo operators, Lichae et al.^27^ studied the effects of antiviral drug medicament on an HIV-1 infection model of CDFP-TC. An approximative solution was derived using Laplace transform and the Adomian decomposition procedure. Based on Srivastava’s research^13^, Ferrari et al.^28^ have created an HIV model using fractional Caputo operators that suggests the possibility of a reverse-transcriptase inhibitor. They showed positive invariance of the model, its existence, and its uniqueness. Also, they examined the stability cases of this model. Researchers are now particularly interested in the qualitative theories for mathematical fractional models. A fractional HIV model using Caputo-Fabrizio operator was discussed by Nazir et al^29^. Using fixed point method, they discovered several requirements for solutions’ existence. For a fractional model of HIV-TB built on Mittag-Leffler formulation, Khan et al.^30^ researched and analyzed several stability and existence discoveries. Additionally, numerical results are attained. In^31^ A fractional HIV infection treated with antiretroviral drug has been presented and examined by Kongson et al. using a fractional generalized Caputo operator.

In the current study, we provide a novel mathematical fractional model for HIV contagion using recently fractional enlarged differential and integral operators. This model includes four nonlinear fractional differential equations with fractional enlarged derivatives. We investigate the existence and uniqueness findings of the suggested fractional HIV model using Banach’s and LSNA fixed point theorems. Additionally, we look into several U-S types for the offered HIV fractional model. Furthermore, we demonstrate how the attained may be compared to certain outcomes from other earlier published works.

## Principal tools

### Fractional enlarged operators

The current section covers definitions, concepts, and key discoveries of the fractional enlarged operators, that will be employed during this study.

#### Definition 2.1

^32^ Assume $\rho \in (0,1]$, $\eta$ belongs to $\mathbb {C}$, and $\text {Re}(\eta )>0$. The modified integral fractional operator of the function *A* is recognized by:

1 $$\begin{aligned} \left( ^{\eta }\mathbb {I}^{\rho }_\vartheta A\right) (t)=\frac{1}{\Gamma (\eta )}\int _0^t\Psi ^{\eta -1}(t,\varkappa ,\rho )\frac{A(\varkappa )}{\vartheta (\varkappa ,\rho )}d\varkappa ,\ \ \ t\ge 0, \end{aligned}$$

where $\Gamma$ indicates the typical Gamma formula, $\Psi (t,\varkappa ,\rho )=\int _\varkappa ^t\frac{dv}{\vartheta (v,\rho )}$ and $\vartheta$ depends continuously on $(t,\rho )\in \mathbb {R}_+\times (0,1]$. Also, $\vartheta (t,1)$ equals one for all *t* in $\mathbb {R}_+$, $\vartheta (t,\rho )\ne 0$ for all $t\in \mathbb {R}_+$, $\rho \in (0,1]$ and $\vartheta (.,\rho _1)\ne \vartheta (.,\rho _2)$ whenever $\rho _1\ne \rho _2$.

In^33^, the authors proposed a new fractional enlarged integral operator as follows:

2 $$\begin{aligned} _{\varrho ,p}\mathbb {I}^\rho A(t)=\int _0^t\frac{(\varkappa -p(1+\varrho _p(\varkappa ,\rho ))A(\varkappa )}{(\varkappa -p)\varrho _p(\varkappa ,\rho )}d\varkappa , \quad t\in \mathbb {R}_+, \end{aligned}$$

where $\varkappa \ne p\in \mathbb {R}$ and $\varrho _p:\mathbb {R}_+\times (0,1]\rightarrow \mathbb {R}$ is a continuous mapping with the following features:

- $$\varrho _p(t,1)=1$$;   $$t\in \mathbb {R}_{+},$$
- $$\varrho _p(t,\rho )\ne 0$$;   $$t\in \mathbb {R}_{+},\ \rho \in (0,1]$$,
- $$\varrho _p(.,\rho _1)\ne \varrho _p(.,\rho _2)$$;   $$\rho _1,\rho _2\in (0,1]$$,
- $$\varrho _0(t,\rho )=\vartheta (t,\rho )$$;   $$t\in \mathbb {R}_{+},\ \rho \in (0,1]$$.

We may repeat the integral (2) *k* times using the Cauchy formula for sequential integrals, and the outcome is as follows.

3 $$\begin{aligned}{} & {} {}^k_{\varrho ,p}\mathbb {I}^{\rho }A(t)=\int _0^t\frac{(\varkappa _1-p(1+\varrho _p(\varkappa _1,\rho ))d\varkappa _1}{(\varkappa _1-p)\varrho _q(\varkappa _1,\rho )}\int _0^{\varkappa _1}\frac{(\varkappa _2-p(1+\varphi _p(\varkappa _2,\rho ))d\varkappa _2}{(\varkappa _2-p)\varrho _q(\varkappa _2,\rho )}\nonumber \\{} & {} \quad ...\int _0^{\varkappa _{k-1}}\frac{(\varkappa _k-p(1+\varrho _q(\varkappa _k,\rho ))A(\varkappa _k)}{(\varkappa _k-p)\varrho _p(\varkappa _k,\rho )}d\varkappa _k\nonumber \\{} & {} \quad =\frac{1}{\Gamma (k)}\int _{0}^{t} {_p{\Psi }^{k-1}}(t,\varkappa ,\rho )\frac{(\varkappa -p(1+\varrho _p(\varkappa ,\rho ))A(\varkappa )}{(\varkappa -p)\varphi _p(\varkappa ,\rho )}d\varkappa , \end{aligned}$$

where

4 $$\begin{aligned} {}{_p{\Psi }}(t,\varkappa ,\rho )=\int _\varkappa ^t\frac{(u-p(1+\varrho _p(u,\rho ))}{(u-p)\varrho _p(u,\rho )}du. \end{aligned}$$

We define the fractional enlarged integral by changing the positive integer *k* to a complex number $\eta$.

#### Definition 2.2

^34^ The fractional enlarged integral of a function *A* is given by

5 $$\begin{aligned} ^\eta _{\varrho ,p}\mathbb {I}^{\rho }A(t)= \frac{1}{\Gamma (\eta )}\int _{0}^{t} {_p{\Psi }^{\eta -1}} (t,\varkappa ,\rho )\frac{(\varkappa -p(1+\varrho _p(\varkappa ,\rho ))A(\varkappa )}{(\varkappa -p)\varrho _p(\varkappa ,\rho )}d\varkappa , \end{aligned}$$

where $\eta \in \mathbb {C}$ with $\text {Re}(\eta )>0$ and $_p\Psi$ is given by Eq. (4).

#### Definition 2.3

^34^ The fractional enlarged derivative of the function *A*, in Riemann-Liouville style, is given by:

6 $$\begin{aligned} \left( ^{\eta }_{\varrho ,p}\mathbb {D}^{\rho } A\right) (t)= & {} Z^k\left( ^{k-\eta }_{\varrho ,p}\mathbb {I}^{\rho }y\right) (t)\nonumber \\= & {} \frac{Z^k}{\Gamma (k-\eta )}\int _{0}^{t} {_p{\Psi }^{k-\eta -1}} (t,\varkappa ,\rho )\frac{(\varkappa -p(1+\varrho _p(\varkappa ,\rho ))A(\varkappa )}{(\varkappa -p)\varrho _p(\varkappa ,\rho )}d\varkappa \ \ \ t\ge 0, \end{aligned}$$

where $$Z=\left( \frac{(\varkappa -p)\varrho _p(\varkappa ,\rho )}{(\varkappa -p(1+\varrho _p(\varkappa ,\rho ))}\right) \frac{d}{dt}$$.

#### Definition 2.4

^32^ The fractional enlarged operator for a function *A*, in Caputo style, is given by:

7 $$\begin{aligned} \left( ^{\eta }_{\varrho ,p}\mathbb {D}^{\rho }_C A\right) (t)=\left( ^{\eta }_{\varrho ,p}\mathbb {D}^{\rho }\left( A(s)-\sum _{j=0}^{k-1}\frac{Z^jA(0)}{j!} \ _p\Psi ^j(s,0,\varrho )\right) \right) (t), \end{aligned}$$

where $k=1+[\text {Re}(\eta )]$. For $\eta \in (0,1)$, we obtain

8 $$\begin{aligned} \left( ^{\eta }_{\varrho ,p}\mathbb {D}^{\rho }_C y\right) (t)=\left( ^{\eta }_{\varrho ,p}\mathbb {D}^{\rho } \left( A(s)-A(0)\right) \right) (t). \end{aligned}$$

Now, consider the space

$$\begin{aligned} Y^{k,\rho }_{\varrho _{p}}\left( [0,c]\right) =\left\{ A:[0,c]\rightarrow \mathbb {R}:\ ^{k-1}_{\varrho ,p}\mathbb {D}^{\rho }_C A(t)= _{\varrho ,p}\mathbb {I}^{\rho }B(t)+A(0),\ B\in X^\rho \right\} , \end{aligned}$$

where

$$\begin{aligned} X^\rho =\left\{ B:[0,c]\rightarrow \mathbb {R}: \ _{\varrho ,p}\mathbb {I}^{\rho } B(t) \ \text {exists} \ \forall \ t\in [0,c] \right\} . \end{aligned}$$

The following theorems provide an alternate form for the fractional enlarged Caputo operator.

#### Theorem 2.1

^32^ Assume $\rho ,\ Re(\eta ),\ c>0$ and $k=1+[\text {Re}(\eta )]$. For $A\in Y^{k,\rho }_{\varrho _{p}}\left( [0,c]\right)$,the fractional enlarged operator for a function *A*, in Caputo style, is alternatively provided by:

9 $$\begin{aligned} \left( ^{\eta }_{\varrho ,p}\mathbb {D}^{\rho }_C A\right) (t)=\frac{1}{\Gamma (k-\eta )}\int _{0}^{t} {_p{\Psi }^{k-\eta -1}} (t,\varkappa ,\rho )\frac{(\varkappa -p(1+\varrho _p(\varkappa ,\rho ))\left( Z^k A\right) (\varkappa )}{(\varkappa -p)\varrho _p(\varkappa ,\rho )}d\varkappa . \end{aligned}$$

#### Theorem 2.2

^32^ Assume $\rho ,\ Re(\eta ),\ c>0$ and $k=1+[\text {Re}(\eta )]$. For $A\in Y^{k,\rho }_{\varrho _p}\left( [0,c]\right)$,the following equality holds:

10 $$\begin{aligned} \left( ^{\eta }\mathbb {I}^{\theta }_\xi \ ^{\eta }_{\varrho ,p}\mathbb {D}^{\rho }_C A\right) (t)=A(t)-\sum _{j=0}^{k-1}\frac{(Z^jA)(0)}{j!} \ _p\Psi ^j(t,0,\rho ). \end{aligned}$$

Particularly, If $\eta \in (0,1)$, we obtain

11 $$\begin{aligned} \left( ^{\eta }\mathbb {I}^{\theta }_\xi \ ^{\eta }_{\varrho ,p}\mathbb {D}^{\rho }_C A\right) (t)=A(t)-A(0). \end{aligned}$$

### Model characterization

The current study is based on the HIV models given in^13,28,31^, which are considered an antiretroviral treatment of reversed transcriptase antagonists. The next are the unknown parameters that the model might include:

*I*(*t*): is the overall number of CDFP-TC that are susceptible.
*J*(*t*): represents the number of CDFP-TC infected before reverse transcription (pre-RT division).
*L*(*t*): is the total number of CDFP-TC that are infected and finished reverse transcription (post-RT division) and eligible to produce the virus.
*M*(*t*): indicates the virus’s density.
$$\gamma$$:
denotes the CDFP-TC inflow rate.
*l*: is the CDFP-TC interaction-infection rate.
$$\delta _1$$:
represents the typical CDFP-TC rate of death.
$$\epsilon$$:
is the RT inhibitor’s potency ($0<\epsilon <1$).
$$\varsigma$$:
is the quantity of infected CDFP-TC that are transmitted from pre-RT division to pose-RT division.
*a*: Tmeasures how quickly infected cells revert to their uninfected state as a result of inadequate reverse-transcription.
$$\delta _2$$:
is the death rate for the infected CDFP-TC.
$$\mathfrak {D}$$:
represents the death rate for CDFP-TC that are actively infected.
*S*: is the total number of virus particles produced by the infected CDFP-TC.
*b*: is the virus’s clearance rate.

We examine the following HIV model infection with CDFPP-TC in light of all the aforementioned characteristics and functions.

12 $$\begin{aligned} {\left\{ \begin{array}{ll} \left( ^{\eta }_{\varrho ,p}\mathbb {D}^{\rho }_C I\right) (t)=\alpha -\beta M(t)I(t)-\delta _1 I(t)+(\epsilon \varsigma +a)J(t),\\ \left( ^{\eta }_{\varrho ,p}\mathbb {D}^{\rho }_C J\right) (t)=\beta M(t)I(t)-(\delta _2 +\varsigma +a)J(t),\\ \left( ^{\eta }_{\varrho ,p}\mathbb {D}^{\rho }_C L\right) (t)=(1-\epsilon )\varsigma J(t)-\mathfrak {D} L(t),\\ \left( ^{\eta }_{\varrho ,p}\mathbb {D}^{\rho }_C M\right) (t)=S\mathfrak {D} L(t)-b M(t). \end{array}\right. } \end{aligned}$$

where $^{\eta }_{\varrho ,p}\mathbb {D}^{\rho }_C$ is the recent improved fractional derivative in Caputo form. For easy procedures, consider model (12) as the next shape:

13 $$\begin{aligned} {\left\{ \begin{array}{ll} \left( ^{\eta }_{\varrho ,p}\mathbb {D}^{\rho }_C I\right) (t)=\Sigma _1(t,I,J,L,M)\\ \left( ^{\eta }_{\varrho ,p}\mathbb {D}^{\rho }_C J\right) (t)=\Sigma _2(t,I,J,L,M),\\ \left( ^{\eta }_{\varrho ,p}\mathbb {D}^{\rho }_C L\right) (t)=\Sigma _3(t,I,J,L,M),\\ \left( ^{\eta }_{\varrho ,p}\mathbb {D}^{\rho }_C M\right) (t)=\Sigma _4(t,I,J,L,M), \end{array}\right. } \end{aligned}$$

where $\Sigma _i(i=1,2,3,4)$ are nonlinear functions given by:

14 $$\begin{aligned} {\left\{ \begin{array}{ll} \Sigma _1(t,I,J,L,M)=\alpha -\beta M(t)I(t)-\delta _1 I(t)+(\epsilon \varsigma +a)J(t),\\ \Sigma _2(t,I,J,L,M)=\beta M(t)I(t)-(\delta _2 +\varsigma +a)J(t),\\ \Sigma _3(t,I,J,L,M)=(1-\epsilon )\varsigma J(t)-\mathfrak {D} L(t),\\ \Sigma _4(t,I,J,L,M)=S\mathfrak {D} L(t)-b M(t). \end{array}\right. } \end{aligned}$$

with the conditions $(I(0),J(0),L(0),M(0))^{\mathbb {T}}=(I_0,J_0,L_0,M_0)^{\mathbb {T}}$ and the superscript $\mathbb {T}$ denotes the transpose.

## Analysis of existence and uniqueness

The examination of the solution to the given HIV model (12) will be done in this part utilizing a variety of fixed point results.

Suppose that $b\in \mathbb {R}^+$, $\mu =(I,J,L,M)^{\mathcal {T}},$ and $\Sigma (t,\mu (t))=(\Sigma _j(t,I,J,L,M))$, $j=1,2,3,4$. Also, Suppose that the Banach space $\mathbb {W}=\mathcal {C}\left( [0,b],\mathbb {R}\right)$ of all functions $\mu$ that are continuous and

15 $$\begin{aligned} \Vert \mu \Vert =\sup _{t\in [0,q]}|\mu (t)|, \end{aligned}$$

where $|\mu (t)|=|I(t)|+|J(t)|+|L(t)|+|M(t)|$ and $I,J,L,M\in \mathbb {W}$. Consequently, the initial value problem for the HIV model (12) might be expressed as follows:

16 $$\begin{aligned} {\left\{ \begin{array}{ll} \left( ^{\eta }_{\varrho ,p}\mathbb {D}^{\rho }_C \mu \right) (t)=\Sigma (t,\mu (t)),\\ \mu (0)=\mu _0, \end{array}\right. } \end{aligned}$$

where $$\mu _0=\left( I_0,J_0,L_0,M_0\right) ^{\mathcal {T}}$$.

HIV model (16) can be equivalently replaced by the integral equation shown below, which relates to Theorem 2.2:

17 $$\begin{aligned} \mu (t)=\mu _0+\frac{1}{\Gamma (\eta )}\int _{0}^{t} {_p{\Psi }^{\eta -1}} (t,\varkappa ,\rho ) \frac{(\varkappa -p(1+\varrho _p(\varkappa ,\rho ))\Sigma (\varkappa ,\mu (\varkappa ))}{(\varkappa -p)\varrho _p(\varkappa ,\rho )}d\varkappa . \end{aligned}$$

It is possible to identify the operator $\mathbb {T}:\mathbb {W}\rightarrow \mathbb {W}$ as

18 $$\begin{aligned} \mathbb {T}(\mu (t)):=\mu _0+\frac{1}{\Gamma (\eta )}\int _{0}^{t} {_p{\Psi }^{\eta -1}} (t,\varkappa ,\rho )\frac{(\varkappa -p(1+\varrho _p(\varkappa ,\rho ))\Sigma (\varkappa ,\mu (\varkappa ))}{(\varkappa -p)\varrho _p(\varkappa ,\rho )}d\varkappa . \end{aligned}$$

In the light of this, the corresponding HIV model (16) has a singleton solution if $\mathbb {T}$ has a fixed point.

Now, A nonlinear alternative fixed point theory called Leray–Schauder, is employed to illustrate that a solution to the HIV model (12) exists.

### Theorem 3.1

If the two prerequisites listed below are satisfied:

($C_1$) $\exists$ a function $\mathcal {X}:[0, \infty ) \rightarrow$ $[0, \infty )$ that is continuous and nondecreasing, and another function $\theta \in \mathcal {C}\left( [0,T], \mathbb {R}^{+}\right)$, such that

19 $$\begin{aligned} {\left\{ \begin{array}{ll} \mathcal {X}(l \mu ) \le l \mathcal {X}(\mu ),&{} for all \quad l \ge 1 \quad and \quad \mu \in \mathbb {W},\\ \Vert \Sigma (t, \mu (t))\Vert \le \theta (t) \mathcal {X}(\Vert \mu (t)\Vert ) &{} for each \quad (t, \mu ) \in [0, T] \times \mathbb {R}^4. \end{array}\right. } \end{aligned}$$

($$C_2$$) $$\exists$$ $$G,E>0$$ such that

20 $$\begin{aligned} \left\| \mu _{0}\right\| +\frac{\theta _{0} \mathcal {X}(G) _p\Psi ^{\eta }(T,0,\rho )}{ \Gamma (\eta +1)}<E. \end{aligned}$$

where $\theta _{0}=\sup _{t \in [0, T]}\{\theta (t)\}$.Then, there is one solution to the IVP problem (16), which is identical to the model (12), exists on [0, *T*].

### Proof

Choose a real number $\omega >0$ such that the collection $\bar{\mathbb {B}}_{\omega }=\left\{ \mu \in \mathbb {W}:\Vert \mu \Vert \le \omega \right\}$ is a bounded ball in $\mathbb {W}$. Given the condition $(C_1)$ with $t\in [0,T]$, we obtain

21 $$\begin{aligned} |(\mathbb {T} \mu )(t)|\le & {} \left\| \mu _{0}\right\| +\frac{1}{\Gamma (\eta )} \int _{0}^{t}{_p{\Psi }^{\eta -1}} (t,\varkappa ,\rho )\frac{(\varkappa -p(1+\varrho _p(\varkappa ,\rho ))||\Sigma (\varkappa ,\mu (\varkappa ))||}{(\varkappa -p)\varrho _p(\varkappa ,\rho )}d\varkappa \nonumber \\\le & {} \left\| \mu _{0}\right\| +\frac{\theta _{0} \mathcal {X}(\Vert \mu \Vert ) _p\Psi ^{\eta }(T,0,\rho )}{ \Gamma (\eta +1)}<E, \end{aligned}$$

hence,

22 $$\begin{aligned} \Vert \mathbb {T} \mu \Vert \le \left\| \mu _{0}\right\| +\frac{\theta _{0} \mathcal {X}\left( \omega \right) _p\Psi ^{\eta }(T,0,\rho )}{ \Gamma (\eta +1)}<E. \end{aligned}$$

Thus, each bounded sphere in $\mathbb {W}$ is converted to a bounded sphere via the operator $\mathbb {T}$.

Take $m_1,m_2\in [0,T]$ such that $m_1<m_2$ and $\mu \in \mathbb {B}_{\omega }$, we get

23 $$\begin{aligned}&\left| (\mathbb {T} \mu )\left( m_{2}\right) -(\mathbb {T} \mu )\left( m_{1}\right) \right| \nonumber \\&\quad = \left| \frac{{1}{\Gamma (\eta )}}{\int }_{0}^{m_{2}}{_p{\Psi }^{\eta -1}}(m_2,\varkappa , \rho ) \frac{(\varkappa -p(1+\varrho _p(\varkappa ,\rho ))\Sigma (\varkappa ,\mu (\varkappa ))}{(\varkappa -p)\varrho _p(\varkappa ,\rho )} d\varkappa \right. \nonumber \\&\qquad \left. -\frac{1}{\Gamma (\eta )} \int _{0}^{m_{1}} {_p{\Psi }^{\eta -1}}(m_1, \varkappa , \rho ) \frac{(\varkappa -p (1+\varrho _p(\varkappa ,\rho ))\Sigma (\varkappa ,\mu (\varkappa ))}{(\varkappa -p)\varrho _p(\varkappa ,\rho )}d\varkappa \right| \nonumber \\&\quad \le \frac{\theta _{0} \mathcal {X}(\Vert \mu \Vert )}{\Gamma (\eta +1)}\left( \left| {_p{\Psi }^{\eta }}(m_2,0,\rho )- {_p{\Psi }^{\eta }} (m_2,m_1,\rho )- {_p{\Psi }^{\eta }}(m_1,0,\rho )+ {_p{\Psi }^{\eta }}(m_2,m_1,\rho )\right| \right) \rightarrow 0\nonumber \\&\qquad \text {as}\,\, m_1\rightarrow m_2. \end{aligned}$$

In the light of this, every bounded set can be converted into an equicontinuous set in $\mathbb {W}$ by using the operator $\mathbb {T}$. Additionally, according to the Arzel-Ascoli formula, the operator $\mathbb {T}$ is continuous completely. Take $\mu \in \mathbb {W}$ such that $\mu$ is a solution to $\mu =k\mathbb {T}(\mu )$ with $0<k<1$. Then, $\forall \,m\in [0,T]$, we obtain

24 $$\begin{aligned} |\mu (m)|=|k\mathbb {T} (\mu (t))| \le \left\| \mu _{0}\right\| +\frac{\theta _{0} \mathcal {X}(\Vert \mu \Vert ) {_p{\Psi }^{\eta }}(T,0,\rho )}{ \Gamma (\eta +1)}. \end{aligned}$$

Thus, we have

25 $$\begin{aligned} \Vert \mu \Vert \le \left\| \mu _{0}\right\| +\frac{\theta _{0} \mathcal {X}(\Vert \mu \Vert ) _p\Psi ^{\eta }(T,0,\rho )}{ \Gamma (\eta +1)}. \end{aligned}$$

Using condition $(C_2)$, we obtain that $\Vert \mu \Vert \ne G$. Take $\mathbb {U}:=\{\mu \in \mathbb {W}:\Vert \mu \Vert <G\}$. Since $\mathbb {T}:\bar{\mathbb {U}}\rightarrow \mathcal {W}$ is completely continuous. In view of the collection $\mathbb {U}$, for some $0<k<1,$ there is no $\mu \in \partial \mathbb {U}$ with $\mu =k\mathbb {T}\mu$. Then, using the (LSNA) theorem, we come to the conclusion that the HIV model under investigation has one solution on [0, *T*]. So, the result is now proved. $\square$

Now, The uniqueness of the solution to the under-studied HIV model is demonstrated in the following theorem utilizing the BFP formula.

### Theorem 3.2

Postulate that the continuous function $\Sigma :[0,T]\times \mathbb {W}\rightarrow \mathbb {R}^4$ meets the coming provision.

26 $$\begin{aligned} (C_3): \,\,\, \,\,\,\Vert \Sigma (t,\mu _1(t))-\Sigma (t,\mu _2(t))\Vert \le \mathfrak {K}_{\Sigma }\Vert \mu _1(t)-\mu _2(t)\Vert ,\,\,\,\,\,\forall \mu _1,\mu _2\in \mathbb {W},\,t\in [0,T]. \end{aligned}$$

for some constant $\mathfrak {K}_\Sigma$ that fulfills

27 $$\begin{aligned} {\mathfrak {K}_\Sigma \, _p\Psi ^{\eta }(T,0,\rho )}<\Gamma (\eta +1). \end{aligned}$$

Then, there is just one solution on [0, *T*] for the HIV model (12).

### Proof

Set $\mathcal {G}=\sup _{t\in [0,T]} \Vert \Sigma (t,0)\Vert <\infty$, and choose $w_1\ge \frac{||\mu _0||\Gamma (\eta +1)+\mathcal {G}{_p{\Psi }^\eta }(T,0,\rho )}{\Gamma (\eta +1)-\mathfrak {K}_{\Sigma }{_p{\Psi }^\eta }(T,0,\rho )}$ such that

$$\bar{\mathbb {B}}_{\omega _1}=\left\{ \mu \in \mathbb {W}:\Vert \mu \Vert \le \omega _1\right\}$$.

$$\forall \mu \in \bar{\mathbb {B}}_{\omega _1}$$, we get

28 $$\begin{aligned}{} & {} | (\mathbb {T}\mu )(t)|\le \Vert \mu _{0}\Vert +\frac{1}{\Gamma (\eta )} \int _{0}^{t} {_p{\Psi }^{\eta -1}} (t,\varkappa ,\rho )\frac{(\varkappa -p(1+\varrho _p(\varkappa ,\rho ))||\Sigma (\varkappa ,\mu (\varkappa ))||}{(\varkappa -p) \varrho _p(\varkappa ,\rho )}d\varkappa \nonumber \\{} & {} \quad \le \Vert \mu _{0}\Vert +\frac{1}{\Gamma (\eta )} \int _{0}^{t}{_p{\Psi }^{\eta -1}}(t,\varkappa ,\rho )\left( ||\Sigma (\varkappa ,\mu (\varkappa ))-\Sigma (u, 0)\right\| +\Vert \Sigma (u, 0)\Vert |)\frac{(\varkappa -p(1+\varrho _p(\varkappa ,\rho ))d\varkappa }{(\varkappa -p)\varrho _p(\varkappa ,\rho )} \nonumber \\{} & {} \quad \le \Vert \mu _{0}\Vert +\frac{\omega _1\mathfrak {K}_{\Sigma } +\mathcal {G}}{\Gamma (\eta )} \int _{0}^{t} {_p{\Psi }^{\eta -1}} (t,\varkappa ,\rho )\frac{(\varkappa -p(1+\varrho _p(\varkappa ,\rho ))d\varkappa }{(\varkappa -p)\varrho _p(\varkappa ,\rho )}\nonumber \\{} & {} \quad \le \Vert \mu _{0}\Vert +\frac{{_p{\Psi }^{\eta }}(T,0,\rho )\left( \omega _1\mathfrak {K}_{\Sigma } +\mathcal {G}\right) }{\Gamma (\eta +1)} \le \omega _1. \end{aligned}$$

This proves that, $\mathbb {T}\bar{\mathbb {B}}_{\omega _1}\subset \bar{\mathbb {B}}_{\omega _1}$.

On the other hand, $\forall \,\mu _{1}, \mu _{2} \in \bar{\mathbb {B}}_{\omega _1}$ for each $u \in [0, T]$, we have

29 $$\begin{aligned}{} & {} \left| \left( \mathbb {T} \mu _{1}\right) (u)-\left( \mathbb {T} \mu _{2}\right) (u)\right| \nonumber \\{} & {} \le \frac{1}{\Gamma (\eta )} \int _{0}^{u} {_p{\Psi }^{\eta -1}}(u,\varkappa ,\rho ) \left( ||\Sigma (\varkappa ,\mu _1(\varkappa ))-\Sigma (\varkappa ,\mu _2(\varkappa ))||\right) \frac{(\varkappa -p(1+\varrho _p(\varkappa ,\rho ))d\varkappa }{(\varkappa -p)\varrho _p(\varkappa ,\rho )}\nonumber \\{} & {} \le \frac{\mathfrak {K}_\Sigma }{\Gamma (\eta )} \int _{0}^{u} {_p{\Psi }^{\eta -1}} (u, \varkappa , \rho ) |\mu _{1}(t)-\mu _{2}(t)| \frac{(\varkappa -p(1+\varrho _p(\varkappa ,\rho )) d\varkappa }{(\varkappa -p)\varrho _p(\varkappa ,\rho )}\nonumber \\{} & {} \le \frac{{\mathfrak {K}_{\Sigma }} {_p{\Psi }^{\eta }}(T,0,\rho )}{\Gamma (\eta +1)}\left\| \mu _{1}-\mu _{2}\right\| . \end{aligned}$$

This leads us to the conclusion that $\mathbb {T}$ is a contraction operator based on equations (32) and (29). Consequently, a single solution for the HIV model (12) exits on [0, T] in view of the (BFP) theorem. $\square$

We can acquire a special case of our findings if we set $p = 0$ in the operators (5) and (10). Applying these operators in the prior theorems and model (12), we obtain the following corollaries, which concern the correct version of the results in^32^.

### Corollary 3.2.1

If the two prerequisites ($C_1$) and ($C'_2$) are satisfied:

where

($$C'_2$$) $$\exists$$ $$G,E>0$$ such that

30 $$\begin{aligned} \left\| \mu _{0}\right\| +\frac{\theta _{0} \mathcal {X}(G) \Psi ^{\eta }(T,0,\rho )}{ \Gamma (\eta +1)}<E. \end{aligned}$$

Then, there is one solution to the model (12) defined by the operator (1), exists on [0, *T*].

### Corollary 3.2.2

Postulate that the continuous function $\Sigma :[0,T]\times \mathbb {W}\rightarrow \mathbb {R}^4$ meets the coming provision.

31 $$\begin{aligned} (C'_3): \,\,\, \,\,\,\Vert \Sigma (t,\mu _1(t))-\Sigma (t,\mu _2(t))\Vert \le \mathfrak {K}_{\Sigma }\Vert \mu _1(t)-\mu _2(t)\Vert ,\,\,\,\,\,\forall \mu _1,\mu _2\in \mathbb {W},\,t\in [0,T]. \end{aligned}$$

for some constant $\mathfrak {K}_\Sigma$ that fulfills

32 $$\begin{aligned} {\mathfrak {K}_\Sigma \, \Psi ^{\eta }(T,0,\rho )}<\Gamma (\eta +1). \end{aligned}$$

Then, there is just one solution on [0, *T*] for the HIV model (12) which derived by operator (1).

## Stability results

The local and global stability of biological models has recently piqued the interest of several researchers^35,36^. In this section, we set up certain necessary conditions for model (12) to satisfy various stability hypotheses. Ulam-Hyers stability (U-HS), extended Ulam-Hyers stability (EU-HS), Ulam-Hyers-Rassias stability (U-H-RS), and so on (EU-H-RS). Before presenting the stability theories, the following definitions must first be stated.

Consider the continuous functions $\chi :[0,T]\rightarrow \mathbb {R}_+$ and the real positive constant $\tilde{\varepsilon }$. To lay the groundwork for the stability definitions, we offer the ensuing inequalities

33 $$\begin{aligned}{} & {} \Vert ^{\eta }_{\varrho ,p}\mathbb {D}^{\rho }_C h(t)-\Sigma (t, h(t))\Vert \le \tilde{\varepsilon }, \quad \forall t \in [0, T], \end{aligned}$$

34 $$\begin{aligned}{} & {} \Vert ^{\eta }_{\varrho ,p}\mathbb {D}^{\rho }_C h(t)-\Sigma (t, h(t))\Vert \le \tilde{\varepsilon } \chi (t), \quad \forall t \in [0, T], \end{aligned}$$

35 $$\begin{aligned}{} & {} \Vert ^{\eta }_{\varrho ,p}\mathbb {D}^{\rho }_C h(t)-\Sigma (t, h(t))\Vert \le \chi (t), \quad \forall t \in [0, T]. \end{aligned}$$

Now, we offer a few prerequisites that are necessary for model (12) to satisfy the (U-HS)and (EU-HS) assumptions. These several types of stability are what we define first.

### Definition 4.1

^37^ The equation (16) is stable under U-HS, if it has a solution $\mu \in \mathbb {W}$ $\forall \, \tilde{\varepsilon }>0$, and the accompanying inequality, along with inequality (33) as well, are both true.

36 $$\begin{aligned} \Vert h(t)-\mu (t)\Vert \le \tilde{\varepsilon }\mathcal {C}_\Sigma , \quad t\in [0,T], \,\,\,\,\forall \,\, h\in \mathbb {W}, \end{aligned}$$

where $$\mathcal {C}_\Sigma =\max \left( \mathcal {C}_{\Sigma _j}\right) ^\mathcal {T},\ \ j=1,2,3,4.$$

### Definition 4.2

^37^ The equation (16) is stable under EU-HS, if it has a solution $\mu \in \mathbb {W}$ , and the accompanying inequality, along with inequality (34) as well, are both true.

37 $$\begin{aligned} \Vert h(t)-\mu (t)\Vert \le \chi (t), \quad t\in [0,T], \,\,\,\,\forall \,\, h\in \mathbb {W}, \end{aligned}$$

where $$\chi =\max \left( \chi _j\right) ^\mathcal {T},\ \ j=1,2,3,4,$$ with $$\chi (0)=0.$$

We now present a crucial feature that can be used to achieve U-HS and EU-HS.

### Lemma 4.1

Consider $\eta >0$ and $\rho \in (0,1]$. If $h\in \mathbb {W}$ is a solution of (33), then *h* fulfills the coming inequality

38 $$\begin{aligned} \left\| h(t)-h_{0}-\frac{1}{\Gamma (\eta )} \int _{0}^{t}{_p{\Psi }^{\eta -1}}(t, \varkappa , \rho ) \frac{(\varkappa -p(1+\varrho _p(\varkappa ,\rho ))\Sigma (\varkappa ,\mu (\varkappa ))}{(\varkappa -p) \varrho _p(\varkappa ,\rho )}d\varkappa \right\| \le \frac{\tilde{\varepsilon } {_p{\Psi }^\eta }(T,0,\rho )}{ \Gamma (\eta +1)}. \end{aligned}$$

### Proof

Using inequality (33), $\exists$ $x\in \mathbb {W}$ such that

39 $$\begin{aligned} \Vert x(t)\Vert \le \tilde{\varepsilon },\,\, \forall \,\, t\in [0,T],\, \ x=\max \{x_1,x_2,x_3,x_4\}. \end{aligned}$$

Hence, we get

40 $$\begin{aligned} {\left\{ \begin{array}{ll} {}^{\eta }_{\varrho ,p}\mathbb {D}^{\rho }_C h(t)=\Sigma (t,h(t))+x(t),&{}t\in [0,T],\\ h(0)=h_0\ge 0. \end{array}\right. } \end{aligned}$$

Applying Theorem 2.2 for (40) we obtain

41 $$\begin{aligned} h(t){} & {} =h_{0}+\frac{1}{\Gamma (\eta )} \int _{0}^{t}{_p{\Psi }^{\eta -1}}(t, \varkappa , \rho ) \frac{(\varkappa -p(1+\varrho _p(\varkappa ,\rho ))\Sigma (\varkappa ,\mu (\varkappa ))}{(\varkappa -p)\varrho _p(\varkappa ,\rho )}d\varkappa \nonumber \\{} & {} +\frac{1}{\Gamma (\eta )} \int _{0}^{t} {_p{\Psi }^{\eta -1}}(t, \varkappa ,\rho ) \frac{(\varkappa -p(1+\varrho _p(\varkappa ,\rho ))x(\varkappa )}{(\varkappa -p)\varrho _p(\varkappa ,\rho )}d\varkappa . \end{aligned}$$

Hence,

42 $$\begin{aligned}{} & {} \left\| h(t)-h_{0}-\frac{1}{\Gamma (\eta )} \int _{0}^{t} {_p{\Psi }^{\eta -1}}(t, \varkappa , \rho ) \frac{(\varkappa -p(1+\varrho _p(\varkappa ,\rho ))\Sigma (\varkappa ,\mu (\varkappa ))}{(\varkappa -p) \varrho _p(\varkappa ,\rho )}d\varkappa \right\| \nonumber \\{} & {} \quad \le \frac{1}{\Gamma (\eta )} \int _{0}^{t} {_p{\Psi }^{\eta -1}}(t, \varkappa , \rho ) \frac{(\varkappa -p(1+\varrho _p(\varkappa ,\rho ))\Vert x(\varkappa )\Vert }{(\varkappa -p)\varrho _p(\varkappa ,\rho )}d\varkappa \nonumber \\{} & {} \quad \le \frac{\tilde{\varepsilon } {_p{\Psi }^\eta }(T,0,\rho )}{ \Gamma (\eta +1)}. \end{aligned}$$

$$\square$$

We are now prepared to demonstrate the U-HS and EU-HS.

### Theorem 4.1

Let $\Sigma (t,\mu (t))$ be continuous. If both (32) and $C_3$ are achieved, then model (12) is stable under U-HS and EU-HS conditions.

### Proof

Choose $h\in \mathbb {W}$ to be a solution of Eq.(33), and $\mu \in \mathbb {W}$ to be a solution of Eq. (16). as claimed by Lemma 4.1 and Eq.(17), we get

43 $$\begin{aligned} \Vert h(t)-\mu (t)\Vert\le & {} \left\| h(t)-\mu _{0}-\frac{1}{\Gamma (\eta )} \int _{0}^{t} {_p{\Psi }^{\eta -1}} (t,\varkappa , \rho ) \frac{(\varkappa -p(1+\varrho _p(\varkappa ,\rho ))\Sigma (\varkappa ,\mu (\varkappa ))}{(\varkappa -p) \varrho _p(\varkappa ,\rho )}d\varkappa \right\| \nonumber \\\le & {} \left\| h(t)-h_{0}-\frac{1}{\Gamma (\eta )} \int _{0}^{t} {_p{\Psi }^{\eta -1}}(t,\varkappa , \rho ) \frac{(\varkappa -p(1+\varrho _p(\varkappa ,\rho ))\Sigma (\varkappa ,h(\varkappa ))}{(\varkappa -p)\varrho _p(\varkappa ,\rho )} d\varkappa \right\| \nonumber \\+ & {} \frac{1}{\Gamma (\eta )} \int _{0}^{t} {_p{\Psi }^{\eta -1}}(t, \varkappa , \rho )\Vert \Sigma (\varkappa , h(\varkappa )) -\Sigma (\varkappa , \mu (\varkappa ))\Vert \frac{(\varkappa -p(1+\varrho _p(\varkappa ,\rho ))}{(\varkappa -p) \varrho _p(\varkappa ,\rho )}d\varkappa \nonumber \\\le & {} \left\| h(t)-h_{0}-\frac{1}{\Gamma (\eta )} \int _{0}^{t} {_p{\Psi }^{\eta -1}}(t,\varkappa , \rho ) \frac{(\varkappa -p(1+\varrho _p(\varkappa ,\rho ))\Sigma (\varkappa ,h(\varkappa ))}{(\varkappa -p) \varrho _p(\varkappa ,\rho )}d\varkappa \right\| \nonumber \\+ & {} \frac{\mathfrak {K}_{\Sigma }}{\Gamma (\eta )} \int _{0}^{t} {_p{\Psi }^{\eta -1}}(t,\varkappa , \rho ) \frac{(\varkappa -p(1+\varrho _p(\varkappa ,\rho ))}{(\varkappa -p)\varrho _p(\varkappa ,\rho )}\Vert h(t)-\mu (t)\Vert d\varkappa \nonumber \\\le & {} \frac{\tilde{\varepsilon } {_p{\Psi }^\eta }(T,0,\rho )}{ \Gamma (\eta +1)}+\frac{\mathfrak {K}_{\Sigma } {_p{\Psi }^\eta }(T,0,\rho )}{ \Gamma (\eta +1)}\Vert h(t)-\mu (t)\Vert . \end{aligned}$$

Hence, $$\Vert h(t)-\mu (t)\Vert \le \mathcal {C}_\Sigma \tilde{\varepsilon }$$, where

44 $$\begin{aligned} \mathcal {C}_{\Sigma }=\frac{\frac{{_p{\Psi }^\eta }(T,0,\rho )}{ \Gamma (\eta +1)}}{1-\frac{\mathfrak {K}_{\Sigma }{_p{\Psi }^\eta }(T,0,\rho )}{\Gamma (\eta +1)}}. \end{aligned}$$

Thus, Eq.(12) is stable under U-HS. On the other hand, by putting $\chi (\tilde{\varepsilon })=\mathcal {C}_\Sigma \tilde{\varepsilon }$ with $\chi (0)=0$ implies that (12) is EU-HS stable. $\square$

### Corollary 4.1.1

Achieving both criteria (32) and $C'_3$ makes the HIV model proposed by Hyder et al.,^32^ stable under U-HS and EU-HS conditions.

### Proof

Placing $p = 0$ in the argument of Theorem 4.1 will allow us to prove this corollary. $\square$

### Definition 4.3

^37^ The equation (16) is stable under U-H-RS, if it is has a solution $\mu \in \mathbb {W}$ $\forall \, \tilde{\varepsilon }>0$, and the accompanying inequality, along with inequality (34) as well, are both true.

45 $$\begin{aligned} \Vert h(t)-\mu (t)\Vert \le \mathcal {M}_\chi \tilde{\varepsilon }\chi (t), \quad t\in [0,T], \,\,\,\,\forall \,\, h\in \mathbb {W}, \end{aligned}$$

where $$\mathcal {M}_\chi >0.$$

### Definition 4.4

^37^ The Eq. (16) is EU-H-RS stable if it has a solution $\mu \in \mathbb {W},$ and the accompanying inequality, along with inequality (35) as well, are both true.

46 $$\begin{aligned} \Vert h(t)-\mu (t)\Vert \le \mathcal {M}_{\chi } \chi (t), \quad \forall \, h\in \mathbb {W},\quad t\in [0,T], \end{aligned}$$

Here, we highlight a key feature that can be used to distinguish between U-H-RS and EU-H-RS.

### Lemma 4.2

If $\eta >0$, $\rho \in (0,1]$, and $h\in \mathbb {W}$ is a solution of (34), and the following condition is achieved

$$(C_4)$$

47 $$\begin{aligned} ^\eta _{\varrho ,p}\mathbb {I}^{\rho } \chi (t) \le \Theta _{\chi } \chi (t)\quad t\in [0,T]. \end{aligned}$$

Where, $\Theta _{\chi }>0$. Then *h* fulfills the next inequality

48 $$\begin{aligned} \left\| h(t)-h_{0}-^\eta _{\varrho ,p}\mathbb {I}^{\rho } \Sigma (t, h(t))\right\| \le \tilde{\varepsilon } \Theta _{\chi } \chi (t). \end{aligned}$$

### Proof

Using inequality (34), $\exists z\in \mathbb {W}$ such that

49 $$\begin{aligned} \Vert z(t)\Vert \le \tilde{\varepsilon } \chi (t), \ z=\max (z_1,z_2,z_3,z_4),\ \forall t\in [0,T]. \end{aligned}$$

Then, we have

50 $$\begin{aligned} {\left\{ \begin{array}{ll} ^{\eta }_{\varrho ,p}\mathbb {D}^{\rho }_C h(t)=\Sigma (t,h(t))+z(t),&{}t\in [0,T],\\ h(0)=h_0\ge 0. \end{array}\right. } \end{aligned}$$

Applying the integral operator for (50) and utilizing (49) we get

51 $$\begin{aligned} \left\| h(t)-h_{0}-^\eta _{\varrho ,p}\mathbb {I}^{\rho } \Sigma (t, g(t))\right\| \le \, _{\varrho ,p}^\eta \mathbb {I}^{\rho }\Vert z(t)\Vert \le \tilde{\varepsilon } ^\eta _{\varrho ,p}\mathbb {I}^{\rho } \chi (t) \le \tilde{\varepsilon } \Theta _{chi} \chi (t). \end{aligned}$$

$$\square$$

U-H-RS and EU-H-RS can now be demonstrated for model (12), as follows.

### Theorem 4.2

The model(12) is stable under U-H-RS and EU-H-RS conditions, if it fulfills conditions $(C_3)$, $(C_4)$ and Eq.(32).

### Proof

By using Eq. (17), inequality, Lemma 4.2, and (35), we get

$$\begin{aligned} \begin{aligned} \Vert h(t)-\mu (t)|&\le \left\| h(t)-\mu _{0}-^\eta _{\varrho ,p}\mathbb {I}^{\rho } \Sigma (t, \mu (t))\right\| \\&\le \left\| h(t)-h_{0}-^\eta _{\varrho ,p}\mathbb {I}^{\rho } \Sigma (t, h(t))\right\| \\&+\frac{1}{\Gamma (\eta )} \int _{0}^{t} {_p{\Psi }^{\eta -1}}(t,\varkappa , \rho ) \frac{(\varkappa -p(1+\varrho _p(\varkappa ,\rho ))}{(\varkappa -p)\varrho _p(\varkappa ,\rho )}\Vert \Sigma (t,h(t))-\Sigma (t,\mu (t))\Vert d\varkappa \\&\le \left\| h(t)-h_{0}-^\eta _{\varrho ,p}\mathbb {I}^{\rho } \Sigma (t, h(t))\right\| \\&+\frac{\mathfrak {K}_{\Sigma }}{\Gamma (\eta )} \int _{0}^{t} {_p{\Psi }^{\eta -1}}(t,\varkappa , \rho ) \frac{(\varkappa -p(1+\varrho _p(\varkappa ,\rho ))}{(\varkappa -p)\varrho _p(\varkappa ,\rho )}\Vert h(t)-\mu (t)\Vert d\varkappa \\&\le \tilde{\varepsilon } \Theta _{\chi } \chi (t)+\frac{\mathfrak {K}_\Sigma {_p{\Psi }^{\eta }}(T,0,\rho )}{ \Gamma (\eta +1)}\Vert h(t)-\mu (t)\Vert \end{aligned} \end{aligned}$$

Therefore,

52 $$\begin{aligned} \Vert h(t)-\mu (t)\Vert \le \mathcal {M}_{\chi } \tilde{\varepsilon } \,\chi (t), \end{aligned}$$

where

53 $$\begin{aligned} \mathcal {M}_{\chi }=\frac{\Theta _{\chi }}{1-\frac{\mathfrak {K}_\Sigma {_p{\Psi }^{\eta }}(T,0,\rho )}{ \Gamma (\eta +1)}}. \end{aligned}$$

Model (12) is therefore stable under U-H-RS condition. Moreover, the model (12) is stable under the EU-H-RS condition when $\tilde{\varepsilon }=1$ in (52) with $\chi (0)=0$. $\square$

### Corollary 4.2.1

The HIV model due to Hyder et al.,^32^ is stable under U-H-RS and EU-H-RS conditions, if it fulfills conditions $(C'_3)$ Eq. (32).

## Conclusion

In the current paper, the prospect of developing a creative mathematical fractional model for HIV infection was considered. Recent enlargements in fractional operators were used to construct this fractional model. The existence and uniqueness findings of this fractional model have been investigated using Banach’s and LSNA fixed point theorems. U-S of various forms is also examined for the suggested fractional HIV model. Comparing the outcomes from the current studies with those from the earlier literature, one may observe that if $p=0,\ _0\varrho (t,\rho )=t^{1-\rho }$, then $_0\Psi (t,\varkappa ,\rho )=\frac{1}{\rho }\left( t^\rho -\varkappa ^\rho \right)$, and the fractional concepts in 2.2 and 2.3 concur with that explored by Jarad et al. in^38^. In this context, the conclusions reached in Theorems 3.1, 3.2, 4.1, and 4.2 match those established in^31^ for HIV infection. Further, if $\rho \rightarrow 1$, results 3.1, 3.2, 4.1 seem to confirm the conventional conclusions about HIV infection that were reached using the standard Newton’s derivative.

## Data Availability

The datasets used and/or analysed during the current study available from the corresponding author on reasonable request.
